# Optimising Nutrition for Sustainable Pig Production: Strategies to Quantify and Mitigate Environmental Impact

**DOI:** 10.3390/ani15101403

**Published:** 2025-05-13

**Authors:** Shane Maher, Torres Sweeney, John V. O’Doherty

**Affiliations:** 1 School of Agriculture and Food Science, University College Dublin, Belfield, D04 W6F6 Dublin, Ireland; shane.maher.1@ucdconnect.ie; 2 School of Veterinary Medicine, University College Dublin, Belfield, D04 W6F6 Dublin, Ireland; torres.sweeney@ucd.ie

**Keywords:** swine, sustainability, life cycle assessment, nutrient utilisation, faba beans, grain preservation, organic acids, microbiota, maternal nutrition

## Abstract

The intensifying global demand for food presents significant challenges for sustainable pig production, particularly in the context of escalating input costs, environmental degradation, and resource scarcity. Life cycle assessment provides a comprehensive framework for quantifying environmental impacts and identifying production hotspots within pig production systems. Feed production and manure management are consistently identified as major contributors, emphasising the need for targeted interventions. Although soybean meal remains a key protein source, its association with deforestation and biodiversity loss is driving an interest in more sustainable alternatives. In temperate climates, faba beans offer a promising, locally sourced option, though their wider adoption is limited by amino acid imbalances and anti-nutritional factors. Grain preservation is another critical consideration, as post-harvest losses and fungal contamination compromise feed quality and animal health. Organic acid preservation has emerged as an energy-efficient, cost-effective alternative to industrial drying, improving storage stability and reducing fossil fuel dependence. Additional nutritional strategies, including dietary crude protein reduction, carbohydrate source modification, feed additive inclusion, and maternal nutritional interventions, can enhance nutrient utilisation, intestinal health, and herd resilience while mitigating environmental impact. This review explores practical feed-based strategies to support sustainable, resilient, and resource-efficient pig production and contribute to global food security.

## Simple Summary

Pig farming plays an important role in global food production but faces growing challenges, including rising input costs, environmental pollution, and increasing pressure on natural resources. Life cycle assessment is a useful tool for measuring the environmental impact of food systems and identifying where emissions originate. Feed and manure are the two main sources of emissions in pig farming. Widely used ingredients like soybean meal are linked to deforestation and biodiversity loss in some regions. In cooler climates, locally grown faba beans offer a more sustainable alternative, though their inclusion requires careful management due to its nutritional limitations. Maintaining feed quality is also crucial, as post-harvest losses and contamination can impact productivity and animal health. Preserving grain with organic acids offers a safe, energy-efficient alternative to traditional grain drying, reducing fossil fuel use while maintaining feed safety. Other dietary strategies, such as lowering protein levels, adjusting fibre sources, using feed additives, and supplementing sow diets, can improve digestion, reduce waste, and minimise environmental pollution. Together, these strategies can offer practical, science-based solutions for making pig farming more sustainable and efficient.

## 1. Introduction

The increasing global demand for food, driven by population growth, longer life expectancy, and improving living standards, presents significant challenges for sustainable agriculture. By 2050, food demand is projected to rise by up to 50% [1,2], placing pressure on agricultural systems to enhance productivity while minimising environmental impact [3]. This pressure is further intensified by climate change, urbanisation, and land use transformation, which constrain the availability of natural resources [4,5]. Both crop and livestock production face growing scrutiny due to their competing demands for land, water, and energy, as well as their contribution to air, water, and soil pollution [6,7]. Pig production plays a central role in global food security, accounting for approximately one-third of total meat consumption [8]. Pork remains a dietary staple, particularly in developing countries, providing high-quality protein and essential micronutrients [9,10]. However, modern pig production faces mounting economic, environmental, and health-related challenges, including high feed and energy costs, resource limitations, and emissions [11]. Additionally, concerns over antimicrobial resistance and feed safety emphasise the urgent need for more responsible production strategies [12,13].

Achieving sustainable pig production requires balancing environmental goals with economic viability, animal performance, and consumer expectations [14]. In this context, life cycle assessment (LCA) is a widely accepted tool for assessing the sustainability of complex systems such as agriculture and food production [15,16,17]. It provides a quantitative framework for evaluating environmental impacts across different production stages [18]. By identifying key environmental ‘hotspots’, LCA supports the development of targeted mitigation strategies to reduce emissions and improve resource efficiency. This holistic approach enables a comprehensive evaluation, making LCA widely applicable in swine production systems [19].

Feed represents the largest economic and environmental cost in pig production, making ingredient selection and formulation critical [19,20,21]. In addition to influencing animal health, welfare, and productivity, feed composition affects farm profitability and environmental impact, particularly with respect to greenhouse gas (GHG) emissions, land use, fossil energy demand, and water quality [22,23]. In swine diets, cereal grains are the primary energy source, while soybean meal (SBM) remains the main protein source. However, SBM production is linked to major environmental and economic issues including deforestation, biodiversity loss, land degradation, transport-related emissions, and reliance on international trade [24,25]. In response, faba beans have received growing attention across Europe as regionally adapted, sustainable alternatives to imported soybean [25,26]. Nevertheless, nutritional limitations, inconsistent supply, and preservation challenges hinder their widespread adoption [27].

With over one-third of global food production lost post-harvest [28,29], improving grain preservation techniques is critical to ensuring feed safety and reducing waste. In temperate climates, the high moisture content of feed commodities increases their susceptibility to mould and mycotoxin contamination, an issue expected to intensify with climate change [30,31]. Industrial drying is the most conventional method used but it incurs high financial and environmental costs due to its reliance on fossil fuels [32,33,34,35]. Despite its widespread use, the environmental impact of grain drying is often overlooked, and more efficient alternatives remain underexplored.

Organic acid preservation has emerged as a cost-effective, energy-efficient alternative that maintains grain quality while reducing the reliance on energy-intensive drying [36,37,38]. Beyond preservation, dietary organic acids also support the reduction in in-feed antimicrobials [39]. Organic acids can enhance digestive function, modulate intestinal microbiota, reduce manure-related pollution, and improve growth performance in pigs, making them a valuable tool for sustainable production [40,41]. Other nutritional strategies, such as reducing crude protein (CP) levels, modifying carbohydrate sources, and using enzyme supplementation, have also shown potential to lower manure-related emissions and odorous compounds by improving nutrient digestibility and microbial balance [42,43]. In addition to direct dietary interventions, maternal nutrition is gaining recognition as a proactive approach to improve offspring health and environmental sustainability. Optimising sow diets during late gestation and lactation can improve piglet microbial colonisation and resilience, reducing post-weaning challenges and antimicrobial reliance [44]. Such interventions have the potential to induce lifetime improvements in digestive health, immune function, and overall production efficiency.

**Figure 1:**
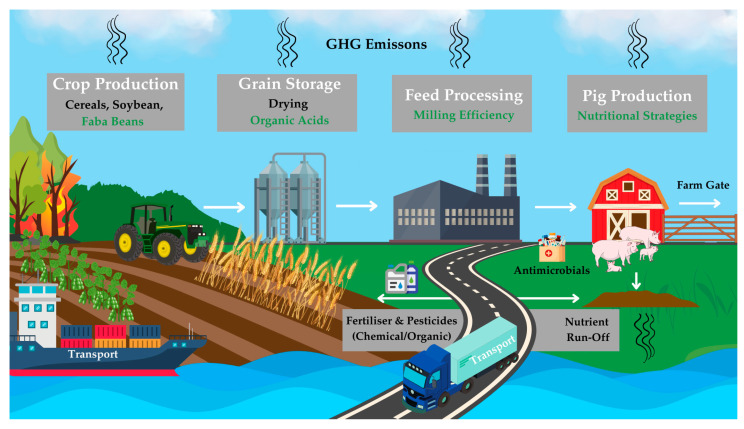
Schematic overview of the pig production chain from crop production to the farm gate, illustrating environmental hotspots and opportunities for intervention. Sustainable strategies include the use of regionally grown faba beans as alternatives to imported soybean, using organic acids for energy-efficient grain preservation, and applying targeted nutritional strategies to enhance gut health, feed efficiency, and nutrient management on farm. These interventions aim to lower greenhouse gas emissions, imported feed dependence, and nutrient losses across the system.

Both European Union (EU) and national policies are increasingly steering the transition towards more sustainable livestock systems by promoting innovation, emission reduction strategies, and improved resource management [45,46]. Science-based nutritional interventions can help producers meet regulatory requirements while maintaining economic viability. This review explores a set of practical, feed-related strategies, each targeting specific sustainability challenges within the pig production chain. Specifically, it focuses on nutritional approaches that reduce nutrient excretion, minimise odorous emissions, and support gut health; the potential of faba beans as sustainable alternatives to SBM; the dual role of organic acids as grain preservatives and functional feed additives; and the impact of maternal nutrition on offspring development and performance. Using an LCA perspective, this review identifies critical environmental hotspots and evaluates how targeted dietary interventions can mitigate these impacts, ultimately supporting a more environmentally and economically sustainable pig sector, as illustrated in Figure 1.

## 2. Life Cycle Assessment in Pig Production

LCA is a standardised methodology used to quantitatively evaluate the environmental impacts of a product across all stages of its life cycle, from raw material extraction to final disposal (cradle-to-grave) [18]. In pig production, LCA has been widely applied to assess GHG emissions, resource use, and other environmental pressures. It helps identify environmental hotspots, compare different feed or production systems, and informs decision making to develop more sustainable practices. As such, LCA is a valuable tool for improving the environmental performance of pig production systems [47,48,49,50,51,52,53].

### 2.1. Methodology

LCA follows internationally recognised standards, including ISO 14040 and ISO 14044 [54,55], and consists of four key phases. The first and arguably more important step is defining the goal and scope, which establishes the study’s objective, the system boundary, the allocation method, and the functional unit (the reference to which all inputs and outputs are scaled). In pig production, 1 kg of live weight at the farm gate is commonly used (Figure 1), although alternative functional units such as carcass weight or protein content have also been reported [56,57,58]. The second phase, life cycle inventory (LCI), involves compiling data on elementary flows such as feed, water, and energy use, as well as emissions, nutrient losses, and waste generation [59]. Where primary farm-level data are unavailable, secondary sources including the literature or databases (Ecoinvent, Agribalyse, and Agri-Footprint) may be used [60,61,62]. However, the variability in databases and data assumptions complicate comparability between studies [20]. The third phase, life cycle impact assessment (LCIA), translates or ‘calculates’ inventory data into impact categories, including global warming potential (GWP; also known as climate change), acidification potential (AP), and eutrophication potential (EP), among others [63]. This phase can be largely automated using software packages such as OpenLCA, GaBi, and SimaPro [64,65,66]. The interpretation is the final stage, which involves testing model sensitivity, identifying key hotspots, and providing recommendations or potential mitigation strategies based on the results obtained [67].

### 2.2. Challenges and Opportunities

Despite its value, the application of LCA in pig production faces several challenges that limit the comparability, transparency, and practical relevance of results [20]. A major issue is the inherent methodological flexibility of LCA. While this adaptability allows tailoring to specific goals, it also introduces variability in system boundaries and functional units [19,68]. For instance, some studies may adopt a cradle-to-farm-gate approach, while others extend to the slaughterhouse gate, leading to differing impact values that complicate cross-study comparisons [20]. Functional units also vary widely, ranging from 1 kg of live weight or carcass weight to 1 kg of pork cuts or even 1 tonne of pig, further reducing comparability [21].

Allocation methods for co-products such as manure, straw, or soybean oil also vary. Although ISO 14044 recommends system expansion as the preferred approach, it is often impractical in agriculture due to data and modelling constraints [69]. Consequently, many studies use allocations based on economic value, mass, or energy. Economic allocation is the most commonly used in swine LCAs, but nearly one-third of studies fail to report the method applied, undermining transparency and reproducibility [19].

The inconsistent treatment of land use change (LUC) is another challenge, particularly for high-impact ingredients like SBM. While climate change was the most frequently reported impact category across 74 pig LCAs, the handling of direct LUC (dLUC) was often unclear [19]. Only six studies reported dLUC separately, seven included it in results, and five explicitly excluded it. Most did not state whether LUC emissions were embedded in background datasets, creating uncertainty and a risk of over or underestimation. When included, dLUC raised climate change impacts by up to 6.5 kg CO_2_-eq/kg live weight, with one study reporting a 470% increase compared with LUC-excluded results [70]. Indirect LUC (iLUC) was even less commonly addressed, appearing in just four studies. These finding emphasise the need for transparent reporting of LUC assumptions to improve the credibility of results.

Data quality and availability remain additional persistent barriers. Primary farm-level data collection is often resource intensive, accounting for up to 80% of the total time and cost in conducting an LCA [71]. Although secondary datasets help address these gaps, they may not fully capture the regional or temporal variation in feed production, manure management, and farming practices, potentially compromising the reliability of the results.

Emerging tools such as artificial intelligence (AI) and machine learning have the potential to improve data processing, predictive modelling, and scenario analysis [72]. However, their integration into agricultural LCAs remains in its infancy and requires further validation to ensure accuracy and reproducibility. Similarly, geographic information systems (GIS) are also being explored to analyse spatial variations in nutrient runoff, land use, and air pollution, which could enable more region-specific mitigation strategies [73]. Additionally, social factors such as labour conditions and animal welfare have historically been underrepresented in LCA models; however, more recent studies have started integrating these aspects for a more holistic sustainability assessment [74,75]. Although these advancements may improve LCA applications in pig production, the inherent complexity and methodological inconsistencies remain challenges that must be addressed to ensure more robust, transparent, and actionable assessments.

### 2.3. Production Hotspots

The livestock sector is responsible for approximately 15% of global anthropogenic GHG emissions, with pork production accounting for around 9% of livestock-related emissions [76]. In the EU, agricultural GHG emissions primarily arise from enteric fermentation (45%), soil management (38%), and manure management (15%) [77]. Unlike ruminants, pigs produce relatively low emissions from enteric fermentation [78]. Instead, the most significant contributors to the environmental impacts of swine systems are feed and manure [19]. Therefore, optimising feed formulations and manure management are essential for reducing the sector’s environmental footprint [79,80].

Feed production alone accounts for more than 70% of the environmental impacts of the pig supply chain, driven by fertiliser and pesticide application, land use and LUC, energy-intensive processing, and the extensive transportation networks of globally sourced raw materials [19,21]. In particular, the environmental and economic burdens associated with SBM and grain preservation highlight the urgent need for more sustainable alternatives [81,82]. It was recently reported that feed-related changes implemented over the past two decades have reduced the GWP of pig production by 20–35% [19], demonstrating the substantial mitigation potential of feed-focused interventions.

Manure management is another major environmental hotspot, contributing to nitrogen and phosphorus losses, eutrophication, acidification, and ammonia volatilisation. Housing systems and manure storage conditions directly influence methane, ammonia, and nitrous oxide emissions, with flooring type, storage methods, and temperature playing key roles in emission rates [83]. While technologies such as slurry additives, anaerobic digestion, and low-emission slurry spreading offer promising mitigation options, this review focuses on nutritional strategies as a practical and preventive approach to reducing nutrient excretion, emissions, and manure odour at the source. Optimising feeding practices and diet composition can minimise nutrient excretion, lower emissions, and significantly improve air, water, and soil quality [84,85].

## 3. Nutritional Strategies for Enhancing Sustainability of Pig Production

### 3.1. Feed Formulation and Ingredient Sourcing

The composition of pig diets is primarily determined by ingredient availability, nutritional value, and economic feasibility, which vary by region and production system [86,87]. Modern feed mills employ mathematical optimisation techniques to formulate least-cost diets while ensuring nutrient balance for optimal growth and feed conversion efficiency [88]. However, recent volatility in raw material prices, geopolitical unpredictability, and increasing scrutiny of the environmental impact of imported feedstuffs have intensified the interest in alternative feed sources [25,26].

Cereal grains, such as maize, wheat, and barley, serve as the primary energy sources in pig diets. Maize is widely used due to its high starch content and digestibility. However, its production is resource intensive, requiring substantial irrigation, fertiliser inputs, and land area, contributing to GHG emissions, eutrophication, and soil degradation [89]. The cultivation of maize in temperate climates is limited by the cooler temperatures and shorter growing season. In addition, it often requires the use of plastic film for crop establishment which increases soil temperature and promotes early growth, further adding to its environmental footprint [90]. As a result, wheat and barley serve as viable alternatives. Wheat provides a more balanced amino acid profile than maize, which is important for the total protein intake of pigs [91]. Barley contains a higher fibre content, which, although beneficial for intestinal health, can inadvertently reduce energy digestibility [92]. Non-starch polysaccharides in wheat and barley, such as arabinoxylans and β-glucans, can lower feed efficiency due to their resistance to enzymatic breakdown [93]. Additionally, phosphorus in cereals is largely bound to phytate, which is poorly digested by pigs, increasing phosphorus excretion and environmental risks. To mitigate these issues, diets are commonly supplemented with exogenous enzymes, which improve digestibility [94], enhance mineral bioavailability and reduce the reliance on non-renewable resources [95].

Soybean meal remains the predominant protein source in pig diets due to its high digestibility, well-balanced amino acid profile, and consistent availability [96]. However, its reliance in Europe presents challenges, including price volatility, environmental degradation, and deforestation associated with large-scale cultivation in South America [97]. Given that feed accounts for the largest share of GHG emissions in pig production, the environmental footprint of SBM has become a key target for mitigation [25]. As concerns around imported feed ingredients intensify, interest is growing in sustainable protein alternatives that offer comparable nutritional value with a lower environmental impact. In response, a variety of novel feed sources such as food waste, insect meal, single-cell proteins, microalgae, and fermented substrates, have been evaluated [26,98,99]. While these options show potential for improving land use efficiency and supporting circular economy principles [25], barriers related to cost, feed safety, regulatory approval, and consumer acceptance limit their widespread adoption [100,101]. In contrast, there is a renewed interest in regionally adapted and agronomically feasible protein crops. Among these, grain legumes offer a practical and sustainable alternative to imported SBM. This review specifically highlights faba beans as a locally available option.

Feed formulation also plays a crucial role in optimising nutrient retention and minimising the environmental burden of manure management. Diet composition influences manure characteristics, influencing nutrient excretion, ammonia volatilisation, and odorous emissions [102]. Strategic modifications to dietary protein levels, carbohydrate sources, and the inclusion of feed additives can improve nutrient utilisation, thereby reducing excretion and minimising environmental impacts.

### 3.2. Nutritional Interventions for Minimising Nutrient Losses, Manure Emissions, and Odour

A key concern in intensive pig production is the inefficient utilisation of dietary nutrients, leading to its excessive excretion in manure. This contributes to ammonia and odorous compound emissions, as well as the eutrophication of water bodies [103]. To mitigate these challenges, precision feeding and nutritional strategies have been developed to optimise intestinal microbial populations, enhancing nutrient absorption while minimising waste output [104,105,106]. Approaches such as optimising dietary CP levels, altering carbohydrate sources, and integrating feed additives offer practical solutions for reducing emissions and improving sustainability in pig production [107]. These strategies not only decrease nutrient losses but enhance feed efficiency and support animal health, promoting a balance between animal welfare, environmental responsibility, and economic viability.

Swine manure has tremendous value as a natural fertiliser for crop production but must be managed to minimise GHG emissions and nutrient leaching [108]. A significant concern is the low nutrient retention efficiency in pigs. Research indicates that only 33% of ingested nitrogen and 37% of ingested phosphorus is retained, with the remainder contributing to environmental pollution [88,109]. Excess nitrogen and phosphorus are particularly problematic, as nitrogen volatilisation leads to ammonia emissions, while both nutrients lead to the eutrophication of water bodies. These environmental concerns necessitate nutritional strategies that enhance both nitrogen retention and phosphorus utilisation, reducing their excretion into the environment.

The ‘ideal protein’ concept, introduced by Cole [110], advocates for lowering dietary CP levels and supplementing with synthetic amino acids to improve nitrogen efficiency. This strategy has been effective in reducing nitrogen excretion without compromising animal performance, although it may increase feed costs due to amino acid supplementation requirements [111]. A reduction of 1% in dietary CP has been associated with a 9% decrease in nitrogen excretion, along with lower AP and EP in LCA models [112]. Similarly, reducing CP from 18.5% to 15.5% in growing pigs improved nitrogen and phosphorus retention, enhanced daily gain, and further reduced AP and EP impacts [113]. Reducing CP from 20% to 12% has also been shown to lower urine production by 25%, leading to a more favourable manure composition with a lower urine-to-faeces ratio [105,107]. Since excess urinary nitrogen is primarily excreted as urea, microbial urease in manure converts it to ammonia, contributing to air pollution [114]. Studies suggest for every 1% reduction in dietary CP, ammonia emissions may be reduced by 8–12%, making CP reduction an effective strategy for mitigating environmental impacts [115,116,117,118,119]. However, care must be taken when formulating low-CP diets, especially for younger pigs, to avoid amino acid deficiencies and ensure growth is not compromised [120,121]. Although reducing nitrogen excretion lowers ammonia emissions, its effect on odorous compounds is inconsistent. Some studies suggest that sulphur-containing amino acids contribute to offensive odours [122,123], increasing manure pH and promoting ammonia volatilisation [124,125]. While CP reduction clearly alters manure composition, its impact on gastrointestinal fermentation and odour production remains an area for further investigation.

Dietary carbohydrate composition also influences intestinal microbial populations, which can affect odorous emissions. For example, substituting wheat with barley can reduce proteolytic metabolites and manure odour due to the presence of β-glucans [126,127]. Similarly, oat-based diets promote beneficial gut bacteria such as *Lactobacillus* and *Bifidobacterium*, altering volatile fatty acid profiles and reducing manure odour emissions [128]. Incorporating fermentable carbohydrates is an effective strategy for modifying the microbiota and reducing manure emissions [129]. Studies have shown that supplementing finisher pig diets with sugar beet pulp increases faecal output while reducing ammonia emissions by stimulating microbial nitrogen incorporation [105]. This lowers manure pH, thereby reducing ammonia volatilisation and subsequently decreasing air pollution [104]. However, fermentation effects vary based on carbohydrate structure. Rapidly fermentable fibres promote volatile fatty acid production and microbial activity, whereas less fermentable fibres primarily increase faecal bulk [130].

The use of feed additives such as enzymes, organic acids, and probiotics can also reduce nutrient excretion and manure-related emissions. Enzyme supplementation improves nutrient digestibility and efficiency, reducing the need for higher protein and phosphorus levels in feed. Phytase increases phosphorus bioavailability, lowering inorganic phosphorus requirements and excretion [95]. Protease and carbohydrase enzymes also enhance protein digestion efficiency, decreasing nitrogen excretion and subsequent ammonia volatilisation [131,132,133,134]. Organic acids also influence nutrient digestibility and fermentation processes by enhancing enzymatic activity, improving protein digestibility, and promoting beneficial microbial populations [135,136,137,138], which will be further discussed in Section 3.6. This reduces the substrates available for microbial proteolysis, thereby minimising ammonia and odorous metabolite production [125,139,140]. Another alternative is the introduction of dietary lactic acid bacteria through *Lactiplantibacillus plantarum* supplementation, which has been reported to improve distal gut microbiota composition, reduce protein-derived odorous compounds, and lower manure emissions [141]. This aligns with the broader understanding that saccharolytic fermentation displaces proteolytic fermentation, reducing noxious gas production while improving gut health [142,143]. While challenges remain in balancing nutrient utilisation and cost effectiveness, ongoing research should apply LCA to evaluate the potential of dietary interventions to enhance environmental sustainability and animal productivity.

### 3.3. Integrating Faba Beans into Pig Diets: Opportunities and Challenges

In response to the plant protein shortage in Europe, grain legume cultivation has expanded considerably over the past 15 years [99]. Over the next decade, EU legume production is projected to increase by more than 80%, reflecting a strong shift towards sustainable protein sources [144]. These trends align with the European Green Deal objectives, which aim to promote regional food autonomy and reduced the reliance on imported soybean. Policy incentives supporting legume cultivation provide farmers with new income opportunities and encourage diversification in agricultural production [145]. This renewed emphasis, and indeed availability in grain legumes, presents a practical approach to enhancing sustainable protein sourcing in livestock diets [146,147,148,149].

Beyond their role in nutrition, grain legumes or pulses such as faba beans, peas, and lupins possess nitrogen-fixing properties, which enhance soil fertility and reduce the dependence on synthetic fertilisers [150,151]. Legumes also help decrease nitrate immobilisation during decomposition, making soil nutrients more readily available compared with cereals [152]. Research has shown that crops sown after grain legumes achieve higher yields with reduced fertiliser inputs [153], benefitting from the disruption of pest, disease, and weed cycles [154]. Unlike SBM, which is predominantly grown in tropical climates, legumes thrive in temperate regions, promoting circular economy principles and reducing transport-related emissions. However, some challenges remain, including variability in protein content, amino acid imbalances, and the presence of anti-nutritional factors (ANFs) [27]. Additionally, the high moisture content of pulses at harvest presents storage challenges, emphasising the need for effective preservation techniques to maintain nutritional integrity.

Faba beans (*Vicia faba* L.), also known as fava beans, field beans, horse beans, or broad beans, are the third most widely cultivated legume globally, after soybeans and peas [155]. Their increasing use in livestock feed is attributed to their high protein content (250–300 g/kg) and favourable amino acid profile [156,157]. Faba beans provide lysine and threonine levels comparable with SBM; however, they are deficient in sulphur-containing amino acids, such as methionine and cysteine, which may necessitate dietary supplementation depending on inclusion rates and overall feed formulation [158].

Furthermore, the various ANFs present in faba beans can impair digestion, reduce nutrient absorption, and affect pig performance. Among these, vicine and convicine are two pyrimidine glycosides which can interfere with red blood cell metabolism and negatively affect growth in pigs. However, the development of low-vicine and convicine cultivars is helping to mitigate these effects [27]. Faba beans also contain protease inhibitors, condensed tannins, and oligosaccharides. The Bowman–Birk inhibitor reduces protein digestibility by interfering with trypsin and chymotrypsin, leading to higher endogenous nitrogen losses [159,160]. Although these inhibitors are heat sensitive and largely inactivated through thermal processing, their presence limits the maximum inclusion rates of raw faba beans in pig diets [148]. Condensed tannins, primarily concentrated in the hulls of coloured flower faba bean varieties, can reduce palatability and protein digestibility by forming complexes with dietary proteins and digestive enzymes [161,162]. Although zero-tannin cultivars exist, they often exhibit poorer agronomic performance, including lower yield stability and reduced frost resistance [163]. Additionally, faba beans also contain high levels of non-digestible oligosaccharides, such as raffinose and stachyose, which are highly fermentable in monogastric animals [164]. While moderate fermentation supports intestinal health, excessive intake can lead to flatulence, loose faeces, or diarrhoea, particularly in younger pigs, thereby limiting their inclusion in early-stage diets [27]. Furthermore, the higher crude fibre content of legumes can lead to increased nutrient excretion, raising concerns related to ammonia emissions and manure management [118].

To reduce the impact of these ANFs and improve the nutritional value of faba beans, a variety of processing techniques have been developed. Dehulling is effective at removing tannin-rich outer layers, while soaking and thermal treatments (e.g., boiling, autoclaving, or roasting), can significantly reduce protease inhibitor and oligosaccharide content [27,165]. However, high-temperature treatments can degrade heat-liable amino acids or trigger Maillard reactions, negatively affecting amino acid availability. Extrusion, a widely used technique in feed processing, enhances protein availability and reduces oligosaccharides through the application of heat, pressure, and force [166]. Nevertheless, despite its potential to modify ANF content, extrusion does not necessarily translate into improved pig performance outcomes when applied to faba beans [37,167]. Germination and fermentation are also effective in breaking down complex compounds and increasing bioavailable protein fractions [168]. However, both methods require the careful control of microbial cultures and may pose implementation challenges at the commercial scale. Moreover, the efficacy of these processing techniques can vary with cultivar, initial bean composition, and the specific processing conditions applied.

Beyond genetic and processing considerations, environmental and agronomic factors such as soil quality, crop husbandry practices, and post-harvest processing also influence the nutritional value of faba beans. Industrial drying is often necessary to reduce moisture content and prevent spoilage during storage. However, drying is energy intensive and expensive, as discussed in Section 3.4 below. Additionally, dried beans can present handling and grinding challenges in feed mills, further affecting feed formulation efficiency. Organic acid preservation has emerged as a promising alternative to conventional drying, offering benefits for digestibility, storage stability, and overall feeding value. Recently, grower–finisher pigs offered organic acid-preserved faba beans exhibited higher feed intake and final body weight compared with those consuming conventional SBM-based diets [37]. Further research is needed to optimise these benefits, particularly by integrating LCA to quantify the environmental implications of substituting SBM with faba beans and replacing conventional drying with organic acid preservation.

While the integration of faba beans presents clear sustainability opportunities, potential trade-offs must be carefully managed. The displacement of conventional crops, variability in nutrient composition, and supply chain inconsistencies pose challenges that require attention. To enhance both performance and sustainability, technologies such as near-infrared spectroscopy (NIR) for real-time nutrient analysis and AI-driven feed formulation tools, are increasingly being utilised [72]. As research advances, faba beans hold strong potential as sustainable alternatives to SBM. However, future research should focus on optimising inclusion levels, improving processing technologies, and integrating LCA to comprehensively evaluate feed sustainability trade-offs. Addressing these challenges will be key to ensuring the successful adoption of faba beans as a viable, regionally produced protein source in pig production.

### 3.4. Importance of Grain Preservation for Feed Sustainability

While advances in plant genetics and agronomic practices have improved crop yields, corresponding efforts in grain preservation have lagged behind [169]. In fact, more than one-third of food produced worldwide is lost during the post-harvest phase due to inefficiencies in storage and preservation [33,170]. Poor post-harvest management not only compromises feed supply but also quality and contamination risks [171]. Thus, effective preservation strategies are essential for maximising production, reducing waste, and minimising resource inputs.

Preserving grain quality is also crucial for maintaining feed efficiency, nutritional integrity, and feed safety in swine nutrition. Effective preservation safeguards the physical, compositional, and sanitary attributes of grains, all of which influence their nutritional contribution to pig diets [172]. Physical properties such as grain size, hardness, and moisture content affect milling efficiency, storage stability, and digestibility; compositional factors including energy, protein, fibre, and mineral content determine nutrient availability; while sanitary conditions, particularly fungal contamination, are critical for feed safety [173].

Moisture control plays a key role in preservation, as high moisture levels promote fungal growth, spoilage, and nutrient degradation [174]. In Europe, cereals, legumes, and oilseeds must be stored below 14%, 15%, and 9% moisture content, respectively [175,176]. However, grains are often harvested at higher moisture levels, necessitating industrial drying to prevent degradation [173]. Fungal contamination remains a significant challenge, particularly under humid conditions. Field fungi, such as *Fusarium* spp., infect crops pre-harvest, while storage fungi, including *Aspergillus* and *Penicillium* spp., proliferate in improperly stored grains [177]. These fungi not only degrade grain quality but also produce mycotoxins, toxic compounds that impair livestock health and performance [178].

Pigs are particularly susceptible to mycotoxins due to their high cereal-based diets and limited detoxification capacity [179,180]. Aflatoxins (AF), produced from *Aspergillus*, cause hepatotoxic and immunosuppressive issues. Trichothecenes such as deoxynivalenol (DON), HT-2, and T-2 toxins, mainly from *Fusarium*, reduce feed intake, damage the gastrointestinal tract (GIT), and may cause vomiting [181,182]. Zearalenone (ZEN/ZEA), also from *Fusarium,* disrupts reproductive function due to its oestrogenic effects, while fumonisins impair liver and kidney function [183]. Ochratoxin A (OTA), produced from *Aspergillus* and *Penicillium*, is nephrotoxic and immunosuppressive, leading to long-term organ damage [184]. Although regulatory guidelines aim to mitigate mycotoxin risks in animal feed, enforcement and monitoring can vary by region [185]. Additionally, climate change is increasing the prevalence of toxin-producing fungi, thereby elevating the contamination risks [31,186].

Biological detoxification methods, including enzymatic degradation and probiotic interventions, offer innovative solutions for mycotoxin mitigation in pig feed. Enzymatic treatments targeting mycotoxin deactivation, such as esterases and oxidoreductases, promote feed safety by neutralising toxic compounds before ingestion [187,188]. Certain microbial strains, such as *Lactobacillus*, *Bacillus*, and *Saccharomyces*, have been shown to degrade mycotoxins or reduce their bioavailability in the GIT [189]. Advancements in mycotoxin-binding agents, including activated clays and yeast-derived products, also contribute to minimising mycotoxin exposure in pig diets [190]. These adsorbents effectively sequester mycotoxins in the GIT, preventing their absorption and subsequent toxic effects. However, their efficacy varies based on mycotoxin structure, diet composition, and intestinal health, emphasising the importance of feed formulation and preservation [191,192].

Industrial grain drying remains the primary method for preventing microbial growth and spoilage in storage, yet industrial dryers are energy intensive and heavily reliant on fossil fuels [32,33,34,35]. Drying lowers water activity, inhibiting microbial metabolism, but improper drying can lead to rehydration, uneven moisture distribution, and nutritional degradation [193,194]. Alternative drying methods, such as natural air drying, offer lower cost solutions but depend on suitable climatic conditions, which can slow drying and increase fungal and pest risks [195]. Solar-assisted drying provides a renewable energy alternative, reducing operational costs and environmental impacts; however, its effectiveness is also limited by weather variability as well as high infrastructure costs [196,197]. Hybrid solar drying systems, which incorporate auxiliary heat sources, may enhance drying efficiency [198] but further research is required to ensure cost effectiveness and consistent grain quality across varying environmental conditions. Given the environmental and logistical drawbacks of conventional drying methods, alternative preservation strategies are gaining interest. Among these alternatives, organic acids preservation has emerged as a promising strategy [199], which will be discussed in the following section.

### 3.5. The Potential of Organic Acids as Grain Preservatives in Sustainable Pig Production

Organic acids and their salts are commonly used food preservatives due to their antifungal and antibacterial properties. These compounds, such as propionic, formic, and lactic acids, disrupt microbial cell membranes, preventing spoilage and reducing the need for energy-intensive drying processes [36]. By lowering pH and suppressing fungal activity, these acids effectively inhibit mould growth, extend shelf life, and reduce contamination risks, making them a viable alternative to traditional drying methods [200,201]. Unlike drying, organic acid preservation does not rely on fossil fuel combustion, providing a more environmentally sustainable solution [34].

The process of preserving grain with organic acids is both straightforward and efficient. At harvest, the grain is transferred into a mixing auger or conveyor, where the acid is applied at a controlled concentration and mixed thoroughly to ensure uniform coverage before storage. This approach preserves grain quality and enables faster transfer into storage, easing pressure on harvest logistics and reducing operational costs. Although early concerns were raised regarding equipment corrosion, acid volatility, and feed palatability, these have been largely mitigated through stabilised formulations. Studies show that concentrated organic acids like acetic and formic acid can corrode stainless steel under certain conditions, particularly at high temperature and concentrations. However, buffered or salt-based formulations improve corrosion resistance by enhancing passivation and limiting metal ion diffusion. These stabilised blends maintain preservation efficacy while improving handling safety, equipment compatibility, and storage stability [41,202].

Beyond storage stability, recent studies have also demonstrated nutritional and performance benefits of organic acid-preserved grain in pig diets, as summarised in Table 1. Preserved grains exhibit higher digestible and metabolisable energy values, leading to improved feed intake and daily gain in pigs [36,203]. Interestingly, pigs consuming preserved grain outperformed those receiving the same acid blend as a direct additive [204], suggesting that the preservation process may enhance the bioavailability and functional efficacy of organic acids. These findings indicate that applying organic acids at harvest may offer greater nutritional advantages than direct supplementation by improving grain quality. One possible hypothesis is that organic acid application at harvest interacts with the grain while it is still biologically active, potentially modifying the structure of key components, such as starch and protein, in ways that enhance digestibility. Acidification at this early stage may also help retain functional compounds and inhibit microbial contamination during storage, contributing to a more favourable nutritional profile. In contrast, when organic acids are added at the feed manufacturing stage, they primarily act as acidifiers rather than influencing the grain’s intrinsic properties. While these mechanisms remain speculative, future studies incorporating microscopy, spectroscopy, or targeted biochemical assays could help clarify the structural and compositional changes induced by early organic acid application. Establishing such effects would provide important insight into the mode of action and long-term nutritional value of acid-preserved grain.

Besides nutritional advantages, organic acid-preserved grains positively influence intestinal health at key production stages. Weaned pigs consuming these grains exhibit enhanced nutrient digestibility and a higher abundance of beneficial bacteria such as *Faecalibacterium*, contributing to an enhanced gut microbial composition and a reduced reliance on in-feed antimicrobials [203,204,205,206]. More recently, a study investigating the lifetime effects of organic acid-preserved grain from creep feed to finisher diets found that pigs offered preserved grain had a higher daily gain, an improved feed efficiency from two weeks post-weaning, and a higher body weight from four weeks post-weaning. These pigs showed increased nutrient digestibility at four weeks post-weaning and at slaughter [207]. They also had a higher carcass weight and increased faecal abundance of *Faecalibacterium* at slaughter, suggesting that the preserved grain may help reduce the days to slaughter and maintain a more beneficial GIT microbiome throughout production. These findings emphasise the dual benefit of organic acid preservation in maintaining feed quality while enhancing lifetime health, resilience, and herd productivity.

Integrating advanced preservation techniques is key to improving grain storage efficiency and minimising environmental impacts. Organic acid preservation provides an opportunity to optimise storage conditions, maintain feed quality, and reduce reliance on fossil fuel drying methods. Future research should incorporate LCA to quantify the environmental trade-offs between different grain preservation methods. Addressing grain storage challenges is essential for ensuring a sustainable and cost-effective feed supply, reducing contamination risks, and supporting long-term productivity in swine production systems.

### 3.6. The Potential of Organic Acids as Functional Feed Additives in Sustainable Pig Nutrition

Amid ongoing environmental and economic pressures, maintaining herd health and productivity remains a critical challenge for the pig industry. The EU ban on zinc oxide and increasing restrictions on in-feed antimicrobials have intensified the urgent need for sustainable nutritional strategies to support animal resilience and reduce reliance on pharmaceutical interventions [12,208]. These shifts are particularly important given that poor health status not only compromises productivity but also increases the environmental footprint of pig production systems [209].

Beyond their preservative role, organic acids have emerged as multifunctional feed additives offering benefits beyond pathogen control. Their antimicrobial activity stems from their ability to penetrate microbial cell membranes in their undissociated form, disrupting cellular function and inhibiting pathogen growth [210,211,212]. In contrast to inorganic acids, organic acids are generally less corrosive and better suited for long-term dietary use, especially when used in buffered or salt forms at appropriate levels [213].

The functional properties of organic acids vary depending on their structure. Short-chain fatty acids (SCFA), including formic, acetic, propionic, and butyric acids, contribute to microbial balance and support epithelial integrity [40]. Medium-chain fatty acids (MCFA), like caprylic and capric acids, exert antimicrobial effects and serve as energy sources [214], while tricarboxylic acids (TCA), such as citric and fumaric acid, assist in metabolism and pH regulation [41]. Recent reviews have highlighted the potential of organic acids to improve intestinal health, nutrient digestibility, and growth performance while reducing nitrogen excretion and environmental emissions [215,216,217,218,219,220,221].

#### 3.6.1. The Role of Organic Acids in Weaner Diets

Organic acids have been extensively studied across all stages of pig production, with their use being particularly prominent during the weaning transition. This critical period is characterised by abrupt dietary changes, immature gut and immune function, and heightened susceptibility to enteric infections [222]. During this time, organic acids contribute to piglet health and performance through several key mechanisms [223]. One of their primary roles is gastric acidification, which is vital in early life when endogenous hydrochloric acid secretion is underdeveloped [224,225]. Post-weaning dietary changes often elevate gastric pH, compromising the stomach’s antimicrobial efficiency and digestive efficiency [226]. Organic acids help restore low pH levels, thereby enhancing protein digestion and reinforcing defence mechanisms of the stomach [227,228].

In addition to acidification, organic acids help modulate the gut microbiota by promoting beneficial bacteria such as *Lactobacillus* and *Bifidobacteria* [229,230], while suppressing pH-sensitive pathogens like *Escherichia* and *Salmonella* [231]. Blended organic acid formulations are often more effective than single-acid supplements, supporting a more resilient microbial community [232]. These microbial shifts are frequently accompanied by reductions in *Enterobacteriaceae* populations and improvements in faecal consistency, both of which are indicators of enhanced gut health [233,234,235].

Organic acids also help alleviate the adverse effects of weaning on intestinal morphology. Weaning typically results in villous atrophy and crypt hyperplasia, which impair nutrient absorption [236,237]. Supplementation with organic acids has been shown to improve villus height and the villus height-to-crypt depth ratio, indicating enhanced epithelial function and nutrient uptake [238,239,240]. Furthermore, organic acids may improve feed palatability, encouraging intake when voluntary consumption is often reduced [217,233,241,242]. However, excessive inclusion rates may have the opposite effect, underscoring the importance of optimised formulations [243].

#### 3.6.2. The Role of Organic Acids in Grower–Finisher Diets

Although most research has focused on weaned piglets, a growing body of evidence supports the efficacy of organic acids in grower–finisher pigs. As pigs mature, the GIT becomes more resilient, reducing susceptibility to dietary and environmental stressors. Nevertheless, organic acids continue to improve digestive efficiency and nutrient absorption during this later production stage [39,40]. In grower–finisher systems, key objectives include maximising growth rates, feed efficiency, and carcass quality while minimising environmental impact and production costs. Notably, European fattening units have been associated with up to ten times higher environmental impacts than weaning units [14], primarily due to the longer finishing period, higher feed intake, and increased manure output [51,244].

Organic acids in older pigs have been shown to enhance the digestibility of protein and amino acids, improve the absorption of key minerals such as calcium, phosphorus, magnesium, and zinc, and reduce nitrogen excretion [216,245,246]. These effects contribute to both better performance and lower nutrient losses, helping to reduce emissions associated with pig production [138]. While the gut microbiota in grower–finisher pigs is generally more stable than that of weaned piglets, organic acids can still promote beneficial microbial shifts that support digestive health and feed utilisation.

Fewer studies have evaluated the effects of organic acids on carcass characteristics or meat quality parameters [218,247]. However, emerging evidence suggests potential benefits, including reduced microbial shedding and improvements in food safety indicators [231,248,249]. These findings highlight the need for further investigation into their broader impacts, such as effects on meat pH, colour, tenderness, water holding capacity, and oxidative stability.

While responses may vary depending on acid type, inclusion level, diet composition, health status, and age, the current evidence supports the use of organic acids as a versatile nutritional strategy for enhancing digestive health, nutrient utilisation, and environmental sustainability across all stages of pig production. A comparative summary of their effects during the weaning, growing, and finishing stages is provided in Table 2.

#### 3.6.3. The Role of Organic Acids in Sow Diets

There is a growing recognition of the critical role that maternal nutrition plays in shaping the development, health, and resilience of offspring both before and after weaning [262]. A sow’s diet can influence foetal growth, colostrum and milk composition, microbial transmission, and immune system development, all of which are essential for neonatal survival and long-term productivity [44,263]. While immediate improvements in piglet growth are not always observed, numerous studies report benefits emerging later in life, particularly during the late post-weaning and finishing stages [264,265]. Despite the logistical challenges of conducting longitudinal experiments, maternal dietary interventions hold significant potential for improving herd health, productivity, and sustainability. Among the various bioactive compounds explored in sow diets, including probiotics, prebiotics, algae, milk products, and yeast derivatives [266,267,268,269,270], dietary organic acids have emerged as a particularly promising strategy and are the focus of this review.

Organic acid supplementation during gestation and lactation has shown multiple benefits for maternal digestive function, immune modulation, and piglet development. These outcomes are particularly relevant during late gestation and lactation, when sows experience heightened nutrient demands [271]. Organic acids have been reported to improve nutrient digestibility, enhance colostrum and milk quality, and suppress pathogenic bacterial populations, making them valuable components for maternal feeding strategies [40]. For instance, citric acid supplementation during late gestation and lactation was found to improve CP, calcium, and phosphorus digestibility, enhancing overall nutrient utilisation [272]. Similarly, blends of organic acid increased dry matter, nitrogen, and gross energy digestibility during reproductive phases [273,274]. Improved nutrient absorption supports energy balance, prevents excessive tissue mobilisation during lactation, and reduces reproductive cycle delays, ultimately enhancing sow longevity and productivity [271].

Organic acids may also support sow metabolic health and lactation performance. Blends containing formic, propionic, and butyric acids, along with ammonium salts, have been shown to increase maternal feed intake, reduce the number of weak-born piglets, and improve litter weaning weight [275]. Furthermore, supplementation with SCFA and MCFA blends reduced body weight loss, enhanced milk production, and improved maternal energy balance [276]. Colostrum and milk not only provide energy and nutrients to piglets but also critical immunoglobulins (Ig), antimicrobial peptides, and prebiotic compounds, which help shape early immune responses and microbial colonisation [277,278]. Some studies have found that organic acids can increase the Ig concentrations in colostrum and milk [272,273], leading to improved piglet plasma Ig levels and reduced pre-weaning mortality [279]. However, these effects may vary based on acid type, inclusion rate, and sow parity, as summarised in Table 3.

Importantly, evidence suggests that maternal organic acid supplementation can influence microbial transfer pathways from sows to piglets. While some microbial exposure may occur in utero via amniotic fluid [280,281], most colonisation takes place postpartum through direct contact with maternal skin, mucosal surfaces, colostrum, milk, and faeces [282]. Since piglets naturally ingest sows faeces in the farrowing environment, modulating the sow’s gut microbiota provides a practical route to shaping early microbial seeding [283,284]. Organic acids have been shown to reduce *Escherichia coli* and increase *Lactobacillus* abundance in sow faeces during farrowing and lactation [273]. These microbial shifts not only enhance sow gut health but also support piglet immune maturation, nutrient absorption, and GIT integrity, which are critical for protection against infections during the challenging post-weaning phase [263,285].

Recent studies have explored advanced delivery methods for organic acids, such as tributyrin (a glycerol ester of butyric acid). Supplementation from day 100 of gestation shortened parturition duration, enhanced dry matter and fat digestibility, and increased milk fat and protein content by day 20 of lactation. Notably, piglets from supplemented sows exhibited reduced diarrhoea and higher plasma levels of IL-10, IL-6, and IgA, indicating improved immune function. Faecal microbial analysis revealed greater microbial diversity, with an increased abundance of *Lactobacillaceae*, *Oscillospiraceae*, and *Christensenellaceae* in sows, and a higher prevalence of *Lactobacillaceae* in piglets [286].

In a similar context, offering sows organic acid-preserved grain (65% propionic acid blend) from day 100 of gestation has recently been shown to deliver multifaceted benefits. Sows fed preserved grain showed increased digestibility of dry matter, nitrogen, neutral detergent fibre, and gross energy. At farrowing, their faecal microbiota had increased *Oscillospiraceae* and *Christensenellaceae,* both of which are associated with enhanced fibre fermentation and gut health [287,288]. Piglets born from these sows had healthier faecal scores during lactation, greater faecal *Lactobacillus* abundance at weaning, and superior post-weaning growth and feed efficiency through to slaughter compared with those from sows fed conventionally dried grain diets [289].

Collectively, these findings emphasise the value of organic acids in sow nutrition. Not only do they enhance maternal digestion and gut health, but they also modulate microbial transfer and improve piglet development. The positive effects observed with direct supplementation has laid the groundwork for more advanced delivery methods. Notably, organic acid preserved grain may offer a holistic strategy that can be extended beyond sows to include creep and finisher diets, supporting a cohesive, farm-wide model for enhancing productivity and sustainability. A selection of studies evaluating organic acids in sow diets and their impact on sow and offspring performance is summarised in Table 3.

**Table 3:** The effects of maternal dietary organic acid inclusion on sow and offspring gut health, digestive function, and growth performance.

Supplementation Period	Organic Acid and Inclusion Level	Parity	Lactation Length	Main Effects on Sow	Main Effects on Offspring	Ref.
48 days (d90 of gestation)	Citric acid (0.5, 1.0, or 1.5%)	3.8	24 days	No effect on ADFI or BW change during lactation. 1.5% increased the CATTD of CP and Ca 1.0 and 1.5% increased serum IgG, IgA, and IgM concentrations. 1.5% increased CP, IgA, and IgM concentrations in colostrum and milk (d 14 post-partum).	No effect on total piglets born/weaned. No effect on mortality, birth weight, or weaning weight.	[272]
41 days (d95 of gestation)	Fumaric, citric, malic, caprylic, and capric acid blend (0.1 and 0.2%)	4.0	21 days	No effect on ADFI, BW loss, or BF change during lactation or wean-to-oestrus interval. 0.2% increased the CATTD of DM, N, and GE. 0.2% increased plasma IgG at weaning. 0.2% increased faecal *Lactobacillus* and reduced *E.coli* at farrowing and weaning.	No effect on total piglets born/weaned. No effect on mortality, growth, or faecal scores during lactation. 0.2% increased plasma immunoglobulin level.	[273]
70 days (d73 of gestation)	Fumaric, citric, malic, caprylic and capric acid blend (0.1 and 0.2%)	3.3	28 days	No effect on ADFI, BW loss, or BF change during lactation. No effect on the CATTD of DM, N or GE. Linear increase in faecal *Lactobacillus* at farrowing and weaning. Linear decrease in faecal *E.coli* at weaning.	No effect on total piglets born/weaned, pre-weaning mortality, or faecal scores during lactation. Linear increase in ADG and weaning weight. Linear increase in faecal *Lactobacillus* and linear reduction in *E.coli* at weaning.	[274]
51 days (d85 of gestation)	Formic, propionic, butyric acid and ammonium salt blend (0.25%)	4.4	21 days	Increased ADFI during lactation however BW and BF were not recorded. No effect on serum antioxidant status. No effect on colostrum or milk composition.	No effect on total piglets born/weaned. Reduced number of low-birth weight piglets (<0.7 kg) but no effect on mortality. Increased litter weight/piglet BW at weaning.	[275]
29 days (d107 of gestation)	Formic, acetic, lactic, citric, propionic, sorbic, caprylic, capric and lauric acid blend (0.1 and 0.3%)	2.6	21 days	0.3% increased ADFI and reduced BW and BF loss during lactation. No effect on the wean-to-oestrus interval. No effect on the faecal microbiota pre-farrowing or post-partum, but *Clostridium perfringens* was reduced on d 7 of lactation.	No effect on total born/weaned. Reduced mummified piglets at birth. Both levels increased piglet ADG during lactation and BW at weaning. No effect on the faecal microbiota on d 7 post-partum or at weaning.	[276]
52 days (d85 of gestation)	Sodium butyrate (0.1%)	3.0	22 days	Increased ADFI during lactation. Reduced wean-to-oestrus interval. Increased fat, CP, IgA, IgG, and IgM concentration in colostrum. Sow microbiota not analysed.	Reduced pre-weaning mortality, diarrhoea incidence, jejunal CD, and the expression of inflammatory cytokines in the colon at weaning. Increased the expression of tight junction proteins in the colon at weaning. Increased colonic microbial diversity and plasma IgA, IgG, and IgM concentrations at weaning.	[279]
35 days (d100 of gestation)	Butyric (Tributyrin 0.05%)	N/A	21 days	Reduced total parturition time No effect on ADFI or BF change during lactation. Increased the CATTD of DM, GE, and EE. No effect on colostrum or milk composition. Increased faecal microbial diversity and the abundance of *Lactobacillaceae*, *Oscillospiraceae* and *Christensenellaceae.*	No effect on total piglets born/weaned, pre-weaning mortality, or growth during lactation. Reduced diarrhoea incidence during lactation. Increased microbial diversity and faecal *Lactobacillus* at weaning.	[286]
N/A Entire cycle	K-diformate and formic acid (0.8 and 1.2%)	3.4	28 days	Reduced BF loss during gestation. Increased ADFI during lactation. No effect on BW or BF change during lactation. 1.2% inclusion increased CATTD of ash and EE. K-diformate tended to increase milk fat.	No effect on total piglets born/weaned. Increased birth and weaning weight.	[290]
32 days (d108 of gestation)	Citric and sorbic acid blend (0.05 or 0.1%)	1.5	25 days	No effect on ADFI or BF change during lactation or wean-to-estrus interval. Linear tendency to reduce lactation BW loss. 0.05 and 0.1% increased the CATTD of DM.	No effect on total piglets born/weaned. No effect on pre-weaning mortality. 0.1% increased piglet ADG and reduced diarrhoea incidence during lactation. 0.1% increased offspring weaning weight.	[291]
Late gestation	Sodium butyrate (0.05% or 0.1%)	3.6	N/A	No effect on ADFI, BW loss, milk composition, or blood clinical chemistry during lactation. IgG and IgA in colostrum tended to increase in supplemented sows.	No effect on total piglets born/weaned, pre-weaning mortality, or growth during lactation. 0.1% increased ADFI, ADG, BW, and G:F increased in offspring PW.	[292]
26 days (d110 of gestation)	Sorbic, formic, acetic, lactic, propionic and MCFA blend (0.3%)	4.8	21 days	No effect on ADFI or BW loss during lactation. Reduced BF loss during lactation. No effect on weaning-to-oestrus interval. Reduced faecal *Streptococcus suis* on d 7 post-partum.	No effect on total piglets born/weaned, or pre-weaning mortality. Increased piglet ADG during lactation. No effect on growth from weaning to d 35 PW. Reduced *Clostridium perfringens* on d 7 PW.	[293]
40 days (d100 of gestation)	Organic acid-preserved grain (65% propionic acid blend)	3.2	26 days	No effect on ADFI, BW loss, or BF change during lactation. Increased the CATTD of DM, OM, N, NDF, and GE. Reduced faecal Proteobacteria and increased *Oscillospiraceae* and *Christensenellaceae* at farrowing.	No effect on total piglets born/weaned, pre-weaning mortality, or growth during lactation. Reduced faecal scores during lactation. Reduced faecal Proteobacteria on d 10 post-partum and increased *Lactobacillus* at weaning. Increased ADG and FCR from weaning to slaughter.	[289]

ADFI, average daily feed intake; ADG, average daily gain; BF, back-fat thickness; BW, body weight; Ca, calcium; CATTD, coefficient of apparent total tract digestibility; CD, crypt depth; CP, crude protein; d, day; DM, dry matter; EE, ether extract; FCR, feed conversion ratio; GE, gross energy; G:F, gain-to-feed ratio; Ig, immunoglobulin; K, potassium; N, nitrogen; NDF, neutral detergent fibre; OA, organic acid; OM, organic matter; PW, post-weaning.

## 4. Conclusions

Sustainable pig production requires an integrated approach that balances productivity, herd health, and environmental responsibility. This review highlights the central role of nutrition-based strategies in achieving these goals, particularly through informed ingredient selection, sustainable preservation techniques, and targeted dietary interventions. Life cycle assessment consistently identifies feed production and manure management as major environmental hotspots in pig systems. Strategies such as replacing soybean meal with locally grown faba beans and substituting energy-intensive drying with organic acid preservation offer practical solutions to reduce greenhouse gas emissions and enhance feed system autonomy. Nutritional interventions that lower dietary crude protein, modify carbohydrate profiles, or include functional feed additives such as organic acids and exogenous enzymes have been shown to improve nutrient utilisation and reduce nitrogen losses, ammonia emissions, and odorous compounds. Among these, organic acids emerge as particularly versatile tools. Their dual role as grain preservatives and functional additives has been shown to improve storage stability, enhance digestive efficiency, support beneficial microbiota, and reduce enteric pathogen load across all production stages. These benefits are especially relevant considering increasing restrictions on in-feed antimicrobials and the ban on zinc oxide. Notably, maternal supplementation with organic acids during late gestation and lactation represents a promising nutritional strategy. The studies reviewed here indicate that this approach can enhance maternal digestibility, modulate sow microbiota, and confer lasting benefits to offspring by supporting microbial colonisation, gut development, and growth performance. This reinforces the value of holistic nutritional management to support long-term productivity and resilience.

Despite these advances, several research gaps remain. A greater integration of life cycle assessment with nutritional trials is needed to better quantify the environmental trade-offs and synergies of feed innovations. Further development and processing of legume-based protein sources such as faba beans is needed to improve digestibility, lower nutritional limitations, and support their wider use as alternatives to soybean meal. Continued work is needed to better understand the mode of action of organic acid-preserved grain. Proposed mechanisms such as enhanced nutrient preservation and increased starch and protein availability require further investigation and validation. Research is also needed to optimise the use of organic acids across all production stages, particularly in older pigs. This includes refining inclusion rates, delivery methods, and acid combinations tailored to animal age, health status, and diet composition. Finally, while maternal organic acid supplementation has shown promising results, its long-term effects on gut development, immune programming, and lifetime growth trajectories remain an area of active investigation and considerable interest. By aligning nutritional innovation with practical application, the industry can move towards a more resilient, environmentally responsible model that ensures long-term food security and economic stability.

## Figures and Tables

**Table 1:** The effects of organic acid-preserved grain on intestinal health, digestive function, and growth performance of pigs.

Production Stage	Organic Acid	Effects on Intestinal Health and Digestive Function	Effects on Growth Performance	Ref.
Exp. 1 and 2: Growing Exp. 3: Weaning	Organic acid-preserved grain (57% formic acid blend)	Exp 1: Increased the diet DE and ME content. Exp. 2: No effect on the CAID of amino acids or CP. Exp. 3: No effect on the CATTD of DM, OM, GE, N, EE, P or Ca.	Exp. 3: Increased ADFI and ADG during d 0–28 PW and final BW on d 28 PW.	[36]
Weaning (7–22 kg)	Organic acid-preserved grain (65% propionic acid blend)	Reduced faecal scores and diarrhoea incidence during d 0–21 PW. Increased the CATTD of DM, OM, N, NDF, and GE on d 21 PW. Increased the CAID of DM, OM, N, and GE on d 35 PW. Reduced ileal *Streptococcus* and increased colonic *Faecalibacterium* on d 35 PW. Reduced colonic BCFA on d 35 PW.	Increased ADFI and ADG during d 0–35 PW and final BW on d 35 PW. Preserved grain outperformed pigs supplemented with ZnO after d 21 PW.	[203]
Weaning (7–21 kg)	Organic acid-preserved grain (65% propionic acid blend)	Reduced ileal *Escherichia* and increased ileal and colonic *Faecalibacterium* on d 10 PW. Increased colonic propionate on d 10 PW.	Increased ADFI and ADG during d 0–35 PW and final BW on d 35 PW.	[204]
Weaning (7–24 kg)	Organic acid-preserved grain (65% propionic acid blend)	Increased ileal *Lactobacillus* and colonic *Faecalibacterium* and *Prevotella* on d 8 PW. Preserved grain increased the CATTD of N in low CP diets (17%) on d 30 PW.	No effect on ADFI but improved FCR during d 0–35 PW and increased final BW on d 35 PW.	[205]
Weaning (7–23 kg)	Organic acid-preserved grain (65% propionic acid blend)	Increased duodenal VH on d 8 PW and tended to increase jejunal VH:CD. Increased the CATTD of DM, OM, N, and GE on d 30 PW. Increased colonic *Prevotellaceae* on d 8 PW.	Increased ADFI during d 15–35 PW and improved FCR during d 0–35 PW.	[206]
Suckling to Slaughter (3–120 kg)	Organic acid-preserved grain (65% propionic acid blend)	Increased the CATTD of DM, OM, N, and GE on d 30 PW and at slaughter. Increased faecal microbial diversity at weaning and d 30 PW, and increased *Faecalibacterium* at slaughter.	No effect on ADFI, but increased ADG to slaughter, improved G:F from d 14 PW, higher BW from d 30 PW, and heavier carcass weight at slaughter.	[207]

ADFI, average daily feed intake; ADG, average daily gain; BCFA, branched-chain fatty acids; BW, body weight; Ca, calcium; CAID, coefficient of apparent ileal digestibility; CATTD, coefficient of apparent total tract digestibility; CP, crude protein; d, day; DM, dry matter; EE, ether extract; FCR, feed conversion ratio; GE, gross energy; G:F, gain-to-feed ratio; N, nitrogen; NDF, neutral detergent fibre; OM, organic matter; P, phosphorus; PW, post-weaning; VH, villus height; VH:CD, villus height-crypt depth ratio.

**Table 2:** The effects of dietary organic acid inclusion on intestinal health, digestive function, and growth performance of pigs.

Production Stage	Organic Acid and Inclusion Level	Effects on Intestinal Health and Digestive Function	Effects on Growth Performance	Ref.
Weaning (7–26 kg)	OA1 (fumaric acid; 0.8–0.2%) OA2 (Ca-formate, Ca-lactate, capric acid and caprylic acid blend; 0.3–0.2%) Combination (OA1 + OA2)	No effect on duodenal morphology or gastric, jejunal, ileal, cecal, or rectal pH on d 14 PW. OA1 reduced jejunal villus height and cecal *E. coli* counts on day 14 PW. OA2 increased the relative weight of the large intestine on d 14 PW. No synergistic effect of combination.	No effect on ADFI, ADG, or FCR during d 0–42 PW.	[224]
Weaning (9–18 kg)	Ca-formate, Ca-lactate, lauric, myristic, and capric acid and citric acid blend (0.3%)	Upregulated the expression of jejunal amino acid transporters (EAAT3, CAT2). Increased plasma IgG concentrations. Increased the CAID of most amino acids. Increased ileal and rectal *Lactobacillus* populations.	Increased ADFI, ADG, FCR, and final BW during d 0–28 PW.	[229]
Weaning (8–32 kg)	OA1 (formic and propionic acid blend; 0.1%) OA2 (formic, propionic and butyric acid blend; 0.2%)	OA2 increased VH in the ileum, while both OA increased jejunal VH:CD on d 35 PW. No effect on CATTD of DM, GE, or CP during d 14 or d 35 PW. OA increased faecal *Bifidobacteria* on d 14 PW.	No effect on ADFI, ADG, or BW during d 0–35 PW. OA1 tended to improve G:F overall.	[230]
Weaning (6–12 kg)	Fumaric, citric, malic, caprylic and capric acids blend (0.2% or 0.4%)	Both levels reduced diarrhoea incidence during d 0–7, 7–14, and 14–21 PW.	Both levels increased ADFI, ADG, G:F, and final BW before and after *E. coli* K88 challenge.	[234]
Weaning (6–13 kg)	Sodium butyrate (0.05 and 0.1%)	0.1% increased villus height in the duodenum, jejunum and ileum and reduced jejunal crypt depth on d 21 PW. 0.1% reduced duodenal/ileal, and colonic *E. coli* and duodenal/ileal *Clostridium*.	0.05% had no effect on performance. 0.1% increased ADFI, ADG, and G:F during d 0–21 PW and final BW on d 21 PW.	[238]
Weaning (9–20 kg)	OA1 (blend of formic, acetic and propionic acids combined with MCFA; 0.3%) OA2 (phenolic compound, slow release C12, target release butyrate, MCFA and OA blend; 0.2%)	OA reduced diarrhoea incidence during d 0–14 PW and d 0–28 PW. OA1 increased serum IgM during d 0–14 PW. OA2 reduced jejunal CD, while both OA increased jejunal and ileal VH:CD on day 28 PW. OA1 increased the CATTD of DM, NDF and ADF during d 14–28 PW. OA2 increased the CATTD of NDF, ADF, and P during d 0–14 PW and EE, and P during d 14–28 PW. OA reduced faecal *E. coli* populations on d 28 PW. OA increased total faecal VFA concentrations, including higher acetic, propionic, and butyric acid on d 28 PW.	OA1 improved FCR during d 0–28 PW. OA2 increased ADG during both d 0–14 and d 14–28 and improved FCR during d 0–28 PW.	[240]
Weaning (7–28 kg)	Formic acid (0.14 or 0.64%)	No effect on gastric mucosa thickness, the number of parietal cells, or stomach weights on d 42 PW. 0.64% increased jejunal microbial diversity on d 42 PW.	Both levels tended to increase ADFI and FCR and increased ADG during d 0–21 PW.	[241]
Weaning (8–18 kg)	Butryic, fumaric and benzoic acid blend (0.5 and 1.0%)	No effect on digesta pH. Tended to have higher duodenal and ileal *Lactobacillus* populations and lower ileal *E. coli.*	Increased ADG and FCR when challenged with *E. coli* K88.	[250]
Weaning (8–16 kg)	OA1 (citric acid; 0.5%) OA2 (formic, propionic, lactic, and phosphoric acids; 0.4%)	OA1 increased serum IgG on d 28 PW. OA reduced faecal *Salmonella* and *E. coli* populations on d 21 and 28 PW. OA increased faecal *Lactobacillus* populations on d 14, 21 and 28 PW.	OA1 reduced ADFI, increased G:F but had no effect on ADG during d 0–28 PW. OA2 reduced ADFI, ADG and G:F.	[251]
Weaning (8–13 kg)	OA1 (phenolic compound, slow release C12, target release butyrate, MCFA and OA blend; 0.2%) OA2 (OA1 (0.2–0.4%) + formic, acetic, lactic, propionic, citric and sorbic acids and salt blend (0.6–0.4%)	No effect on diarrhoea incidence. OA increased serum IgG on d 14 PW and IgA on d 28 PW. OA increased total antioxidant capacity on d 14 and 28 PW. No effect on faecal microbial diversity. OA increased the faecal abundance of Firmicutes and reduced Proteobacteria OA increased the abundance of *Lactobacillus* and *Faecalibacterium*.	OA1 had no effect on ADFI, ADG, FCR or final BW during d 0–28 PW. OA2 improved overall ADG and FCR and final BW on d 28 PW.	[252]
Exp. 1: Weaning (7–24 kg)	OA1 (phenolic compound, slow release C12, target release butyrate, MCFA, and OA blend; 0.2%) OA2 (formic, acetic and propionic acids and MCFA blend; 0.3%) Combination (OA1 + OA2)	OA1 and OA2 reduced diarrhoea index during d 15–17 PW. No effect on gastric, jejunal, or colonic digesta pH. OA2 increased duodenal VH d 28 PW. OA1 increased caecal acetic and propionic acid concentrations on d 28 PW. All OA increased colonic acetic, propionic and butyrate concentrations. OA1 and OA2 increased colonic *Lactobacillus,* while OA2 also reduced colonic *E. coli.*	No effect on ADFI, ADG, FCR or BW during d 0–42 PW.	[253]
Exp. 2: Weaning (7–24 kg)	OA1 (phenolic compound, slow release C12, target release butyrate, MCFA, and OA blend; 0.2%) OA3 (formic acid blend; 0.3%) Combination (OA1 + OA2)	All OA reduced diarrhoea index during d 0–7, 7–14, 14–21, and 0–28 PW. No effect on gastric, duodenal, jejunal, ileal, or colonic digesta pH on d 49 PW. Combination increased ileal VH and acetic and propionic acid concentrations. OA1 and OA3 increased microbial diversity. Combination increased the abundance of *Prevotella* in the colon.	OA had improved ADG and FCR during d 43–49 PW.	
Weaning (6–20 kg)	Sorbic, benzoic, butyric, capric, caprylic, and lauric acid blend (0.2%)	Increased ileal VH:CD on d 15 PW. Tended to increase jejunal and ileal VH on d 30 PW. Increased ileal VH on d 45 PW.	Increased BW on day 30 and 45 PW. Tended to increase ADG during d 0–45 PW.	[254]
Weaning (9–20 kg)	Benzoic acid, Ca-formate, fumaric acid blend (0.15%)	Tended to reduce faecal scores during d 14–21 PW. No effect on gastric, duodenal, jejunal, ileal, cecal, colonic or rectal pH. Increased duodenal VH. Increased the CATTD of CP, EE, Ca, and P on d 28 PW. No effect on duodenal, jejunal, or ileal trypsin or chymotrypsin activity. Increased faecal *Lactobacillus* populations on d 28 PW.	Increased ADG during d 14–28 and d 0–28 PW but no effect on ADFI or G:F.	[255]
Weaning (7–25 kg)	Fumaric, citric, malic, caprylic and capric acids blend (0.1% or 0.2%)	Linear reduction in faecal scores. Increased the CATTD of DM and GE and tended to increase N. Increased faecal *Lactobacillus* populations and reduced *E. coli* and *Salmonella.* Tended to increase faecal *Bifidobacterium* and reduce *Clostridium perfringens.* Reduced faecal ammonia.	No effect on ADFI but improved ADG and G:F during d 0–42 PW.	[256]
Weaning (5–24 kg)	OA1 (formic, acetic acid and ammonium formate blend; 0.2%) OA2 (formic acid, acetic, sorbic, propionic, lactic and citric acids, ammonium formate blend; 0.2%)	OA2 reduced diarrhoea incidence during d 0–14, d 14–49, and d 0–49 PW. OA2 tended to reduce CD and increase VH:CD in the duodenum on d 49 PW. No effect on jejunum lipase, amylase or protease activity on d 14 or d 49 PW.	OA1 increased ADFI and ADG during d 0–14 PW and tended to increase ADG during d 0–49 PW. OA2 had no effect on ADFI, ADG, or FCR overall.	[257]
Growing (19–28 kg)	Benzoic acid (0.5%)	Tended to reduce jejunal pH. Increased trypsin, lipase, and amylase activity in the jejunum after 14 days. Reduced CD and increased VH:CD in the jejunum. Increased the CATTD of DM, GE, CP, and EE.	Increased ADFI, ADG and BW after 14 days.	[239]
Growing (23–50 kg)	Fumaric, citric, malic, caprylic and capric acid blend (0.1% or 0.2%)	No effect on the CATTD of DM, N, or GE, or faecal ammonia during week 6.	Increased ADG during the 6-week period.	[258]
Growing (23–54 kg)	Fumaric, citric, malic, caprylic and capric acid blend (0.1%, 0.2% or 0.4%)	No effect on the CATTD of DM, N, or GE during week 6. 0.2% increased faecal *Lactobacillus* but no effect on faecal *E. coli* during week 6 in any group.	0.2% increased ADG, and G:F during the 6-week period.	[259]
Finishing (48–93 kg)	Fumaric, citric, malic, caprylic and capric acid blend (0.2%)	Reduced faecal pH, ammonia, and acetic acid concentrations. Increased the CATTD of DM, GE, CP and EE in groups without dietary antibiotic supplementation.	Improved G:F over 6 weeks without dietary antibiotic supplementation. Negative effect on G:F in antibiotic supplemented group.	[138]
Finishing (50–117 kg)	Fumaric, citric, malic, caprylic and capric acid blend (0.1% or 0.2%)	No effect on blood serum parameters during week 6 or 12. Linear increase in the CATTD of DM, N, and GE during week 12. Linear reduction in faecal ammonia contents during week 6 and 12. Linear increase in faecal *Lactobacillus* populations during week 6 and reduced faecal *E. coli* during week 6 and 12.	Linear increase in ADG during weeks 0–6, weeks 6–12, and overall. No effect on meat quality parameters (pH, water holding capacity, colour, or drip loss).	[260]
Exp. 1: Weaning (6–22 kg) Exp 2: Grow-Finishing (24–140 kg)	Sodium diformate Exp 1: (0.4%, 0.6%, 0.8%, 1% or 1.2%) Exp 2: (0.25%, 0.5%, or 0.75%)	Exp 1: No effect on faecal DM on d 9 PW. Linear reduction in faecal DM on d 24 PW.	Exp 1: Linear increase in G:F during d 0–24 PW. Exp 2: Linear increase in ADG and ADFI from d 60–93 and 93–117. Linear increase in G:F during d 93–117. Exp 2: No effect on carcass characteristics	[261]

ADFI, average daily feed intake; ADG, average daily gain; BCFA, branched-chain fatty acids; BW, body weight; Ca, calcium; CAID, coefficient of apparent ileal digestibility; CATTD, coefficient of apparent total tract digestibility; CD, crypt depth; CP, crude protein; d, day; DM, dry matter; EE, ether extract; FCR, feed conversion ratio; GE, gross energy; G:F, gain-to-feed ratio; Ig, immunoglobulin; N, nitrogen; NDF, neutral detergent fibre; OA, organic acid; OM, organic matter; P, phosphorus; PW, post-weaning; VH, villus height; VH:CD, villus height-crypt depth ratio.

## Data Availability

Not applicable.
